# Hybrid image of three contents

**DOI:** 10.1186/s42492-019-0036-3

**Published:** 2020-02-10

**Authors:** Peeraya Sripian, Yasushi Yamaguchi

**Affiliations:** 1 SIT Research Laboratory, Shibaura Institute of Technology, Room# 04R31, 3-7-5 Toyosu, Koto-ku, Tokyo, 135-8548 Japan; 2 The University of Tokyo, Bldg. #15, Room #505A, 3-8-1, Komaba, Meguro-ku, Tokyo, 153-8902 Japan

**Keywords:** Hybrid image, Contrast sensitivity, Frequency filtering, Scale-space

## Abstract

A hybrid image allows multiple image interpretations to be modulated by the viewing distance. Originally, it can be constructed by combining the low and high spatial frequencies of two different images. The original hybrid image synthesis was limited to similar shapes of source images that were aligned in the edges, e.g., faces with a different expression, to produce an effective double image interpretation. In our previous work, we proposed a noise-inserted method for synthesizing a hybrid image from dissimilar shape images or unaligned images. In this work, we propose a novel method for adding an image to be seen from a middle viewing distance. The middle-frequency (MF) image is extracted by a special bandpass filter, which generates ringing while extracting only specified frequency bands. With this method, the middle frequency should be perceived as a meaningless pattern when viewed from a far distance and close up. A parameter tuning experiment was performed to determine the suitable cutoff frequencies for designing the filter for the MF image. We found that ringings of a suitable size could be used to make the middle frequency less noticeable when seen from far away.

## Introduction

A hybrid image was introduced by Oliva et al. [1] in 2006. It allows a new paradigm in which a single image can be alternatively interpreted as two different kinds of information, with the modulation of the viewing distance. It can be considered as an ambiguous image. An ambiguous image or a double image is a kind of optical illusion, which is created not only for art but also as an experiment stimulus in the field of psychology [2–4], and for studies on the scene perception in the human brain [5, 6]. A traditional famous optical illusion of a duck/rabbit ambiguous image [7, 8] was initially used by psychologists to point out that visual perception relates to mental activity [9]. Another well-known example of an ambiguous image is the painting of the Holy Roman Emperor Rudolph II as Vertumnus by Giuseppe Arcimboldo. Giuseppe Arcimboldo was known for creating imaginative portrait heads by agriculture products such as fruits or vegetables. For the portrait of Holy Roman Emperor Rudolph II, he arranged images of seasonal vegetables as local information in such a way that the whole collection of vegetables formed a face and body resembling the Roman god of plant life, i.e., the global information of the image. From these kinds of figures, we interpret the global content by integrating local information based on perceptual grouping.

Meanwhile, a hybrid image requires the modulation of a degree of visual angle to see other information that is hidden in the image [10]. The degree of visual angle is relative to the actual viewing distance. Originally, a hybrid image was developed as an experimental stimulus to study the human visual system in terms of spatial frequency [11]. A well-known hybrid image is an image that combines portraits of Einstein and Monroe. The same image can be seen as Einstein when viewed from a close distance, or as Monroe when viewed from a few meters away. It is also possible to demonstrate the changing degree of visual angle from big to small by changing the image’s size instead of changing the viewing distance. In addition, it may be possible to see such a hidden image from far away, with both eyes squinted [12], or through a mobile phone’s camera.

According to multiscale perceptual mechanisms of the human visual system, it is possible to present particular spatial frequency information in the image at a certain viewing distance. A hybrid image, I_HB_, can be synthesized with two input images, I_1_ and I_2_, based on this idea [1],

1 $$ {\mathrm{I}}_{\mathrm{H}\mathrm{B}}={\mathrm{L}}_{\mathrm{p}}\left({\mathrm{I}}_1\right)+{\mathrm{H}}_{\mathrm{p}}\left({\mathrm{I}}_2\right) $$

where L_p_ is a lowpass filter and H_p_ is a highpass filter. According to the contrast sensitivity function [13], a human observer can discriminate a sine-wave grating of frequency g, which is 4 to 6 cycles per degree (CPD) of visual angle, at the lowest contrast. The cutoff frequency C cycles per image used to design the high and lowpass filters, is determined according to the viewing angle θ such that C = θg. Here, θ is the degree of visual angle per image, and it is calculated as

2 $$ \uptheta =\frac{180}{\uppi}{\tan}^{-1}\frac{\raisebox{1ex}{$\mathrm{h}$}\!\left/ \!\raisebox{-1ex}{$2$}\right.}{\mathrm{d}} $$

where h is the image height, and d is the distance from the viewer to the image.

The roles of different spatial frequency bands were examined using hybrid visual stimuli, i.e., hybrid images, by Oliva et al. in refs. [14, 15]. In both works, they discovered that most participants—when presented with visual stimuli for a short time—were oblivious to the fact that they viewed the same image that had two interpretations. In addition, the participants observed different spatial frequencies according to the experimental task [15]. It was assumed from these studies that when viewing a hybrid image, the visual system is often unaware of the other information hidden in an unattended frequency band. Hence, a conventional method for the composition of a hybrid image was introduced. However, the source images were well-aligned in those works, for example, faces with different expressions.

To create a compelling hybrid image, we need to calculate the cutoff frequencies for both spatial frequency images from the CPD, where the sensitivity peaks in the contrast sensitivity function. When two images of different shapes are hybridized with the original method, the unaligned parts cause an ambiguous perception of one image at a distance. Consequently, both images are often perceived at the same time, especially when viewed closely. The effect of this problem can be seen in refs. [11, 16], where the experimenter used hybrid stimuli composed of unaligned images, i.e., different visual scenes. Brady and Oliva [16] found that the low-frequency (LF) information could be seen from almost all viewing distances when the hybrid of different visual scenes (for example, bedroom, forest, and living room) was used as stimulus, which was not the case when using properly aligned images like faces with different emotional expressions.

To create a hybrid image that does not rely on the overlap of the source image’s global spatial scale, we need to maintain the separation of the perception of two spatial frequency images with regard to the viewing distance. The main underlying theory is contrast sensitivity. Because human eyes have limited visual acuity depending on the viewing distance, the high-frequency (HF) image automatically falls off the visible area of the contrast sensitivity function; there should be little to no problem viewing the hybrid image from far away, even when the hybrid image is synthesized from source images that contain different shapes. However, when one looks at the hybrid image closely, the overlapping part of the LF image is visible alongside the HF image. Therefore, the main challenge when synthesizing this type of hybrid image is to maintain the separation of the spatial frequencies when the hybrid image is viewed up close.

Ideally, an edge-alignment-free hybrid image is a hybrid image in which the LF image is perceived as noise or is completely disregarded when viewed closely. To achieve this, we need some HF noises that make the LF image less noticeable but do not deteriorate the perception of the HF image. However, the most challenging point is that this contradicts the findings of critical band masking research. For example, Solomon and Pelli [17] tried to identify the role of the human visual system in the perception of letters and gratings. In their work, they superimposed various spatial frequency noises on the fixed size of a letter image. They found that the same frequency noise worsens the perception of an image, i.e., a letter.

Konishi and Yamaguchi [18] challenged this problem by processing the HF and LF images separately before composing the hybrid image. They introduced the use of noises in the HF image to cover parts of the LF image that were not aligned with the HF image, as well as the contrast reduction method in the LF image. For noises in the HF image, they used ringing artifacts as the by-product of the high spatial frequency extraction with the two-level highpass filter. With this method, noises were produced in a nonrandom manner to prevent an ambiguous perception of the HF image. However, ringing produced by this method has low contrast, especially when the ringing is far from the edges in the HF image. To increase the contrast of ringing throughout the image, work in ref. [18] introduced “local contrast adjustment”. In their local contrast adjustment, an image was first separated into small rectangular blocks. Then, the contrast of each block was enhanced by histogram equalization.

To synthesize an edge-alignment-free hybrid image, it is necessary to make the LF image less noticeable and, at the same time, the HF image more noticeable. We proposed two methods called “noise-inserted method” and “color-inserted method” in ref. [19]. The idea of using noise in a hybrid image came originally from the aforementioned work [18]. We successfully synthesized a hybrid image from unaligned source images and proved that our proposed method could achieve the best separation of the spatial frequencies, when compared with the previous methods by refs. [1, 18] in the experiment [20].

In this work, we employ an adapted version of our previously proposed method to synthesize a hybrid image from three different images. The new kind of hybrid image can be interpreted differently from three different distances: far, middle, and near viewing distances.

To present one image at each distance, we use three different frequency filters, each designed to allow different frequency bands (the low, middle, and high) to pass. In this paper, we discuss mainly the method of extracting frequencies regarding the image seen from the middle distance; appropriate cutoff frequencies for synthesizing a hybrid image of three contents are also investigated.

## Methods

Showing three different contents at three distances is a challenging problem that extended from the previous version of our proposed hybrid image. This time, we must consider the image to be seen at the middle distance, which should not be perceptible from up close and far away. The proposed outline is based on our previous work, with the addition of a middle image extracted by a new type of frequency filter. For thorough understanding, the frequency image to be seen from up close is named “HF image”, the frequency image to be seen from the middle distance is named “middle-frequency (MF) image”, and the frequency image to be seen from far away is named “LF image”.

**Fig. 1:**
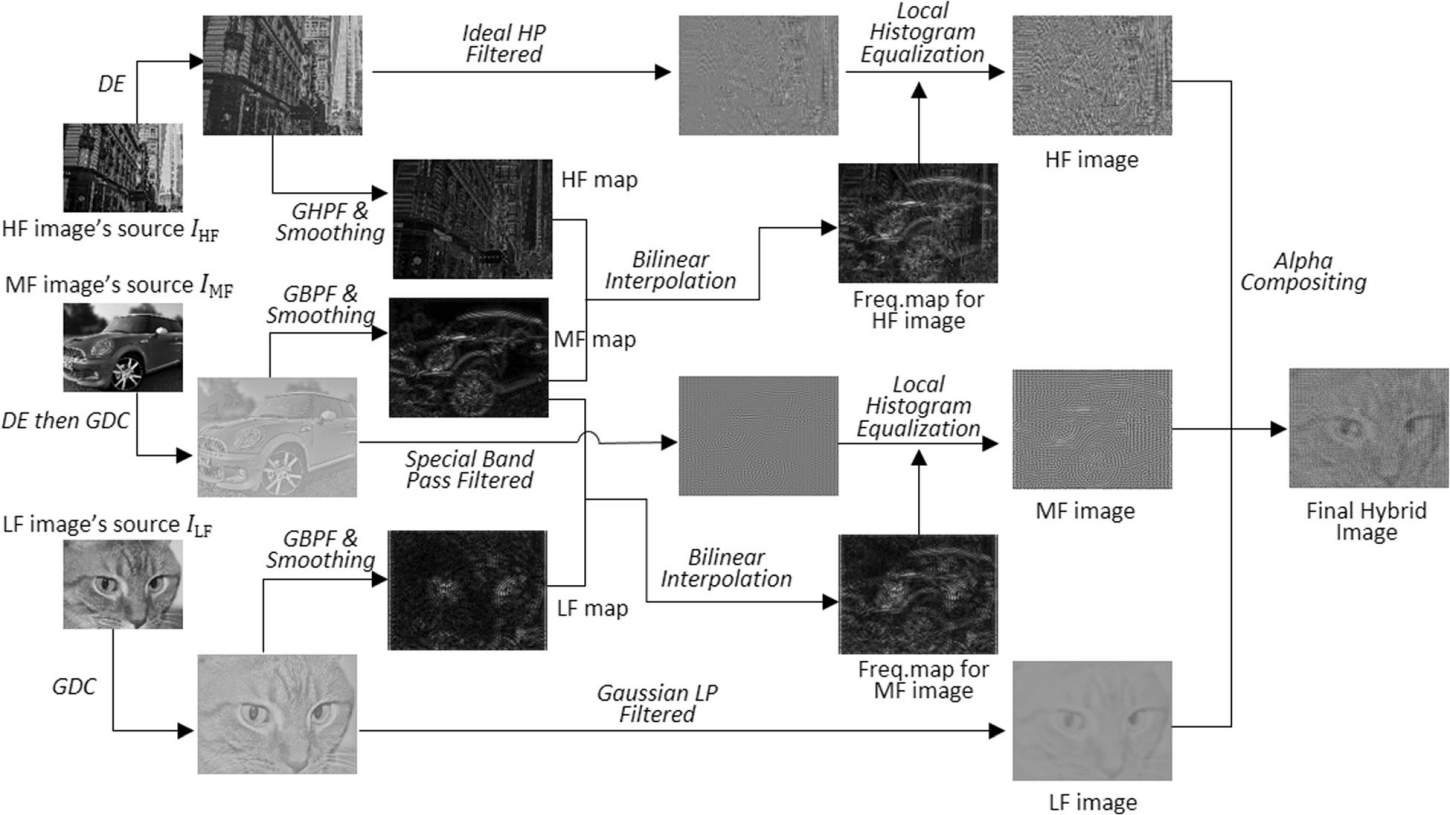
Our proposed algorithm. Source image for MF image: “Maximum Mini,”© 2009 by Christian Senger, used under a Creative Commons Attribution license: [21], and source image for LF image: “Tigger”© 2008 by Jacob Enos, used under a Creative Commons Attribution-ShareAlike license: [22]. HF: High frequency; MF: Middle frequency; LF: Low frequency; GDC: Gradient Domain image range Compression; DE: Detail enhancement; GHPF: Gaussian high pass filtering; GBPF: Gaussian band pass filtering

Similarly to our previous work, we began with the preprocessing of all the source images to achieve the appropriate contrast and details. Then, we extracted each frequency band with different frequency filters in the frequency domain. Finally, we performed local histogram equalization using each frequency image’s local frequency map. The overall process is illustrated in Fig. 1. Source images were taken from refs [21, 22].

### Preprocessing

Different types of preprocessing are performed on each source’s image depending on the distance to be perceived. For instance, we perform Gradient Domain image range Compression (GDC) [23] on the LF source image, I_LF_, to reduce its dynamic range. This reduces the overall contrast so the final image does not stand out too much when synthesized.

For the HF source image, I_HF_, we perform detail enhancement (DE) [24] to enhance existing noises that are difficult to perceive with bare eyes, like digital noises or ISO noises. This way, the HF image extracted from the source image will contain many details. We can cover the presence of the LF image and the MF image by the enhanced details of the HF image.

Because the MF image is inserted between the HF and the LF image, we preprocess the image using the methods from both the HF and the LF images. The source image for the MF image, I_MF_, is firstly preprocessed using DE, and then, the overall dynamic range is compressed using GDC.

### Extraction of frequencies

**Fig. 2:**
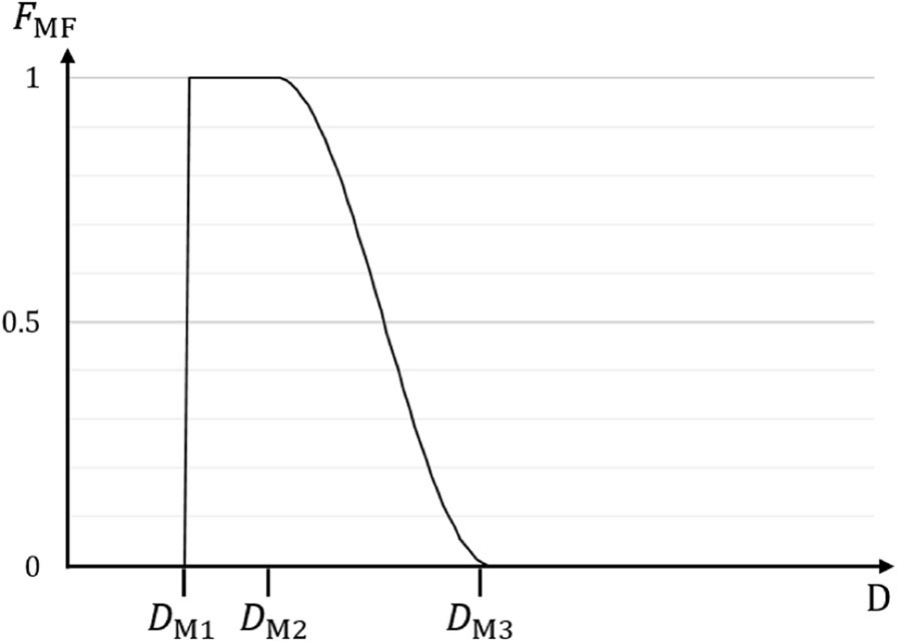
Filter edge profile for the proposed MF filter. MF: Middle frequency

The extraction of frequencies on all three images is performed in the frequency domain. We use a two-level highpass filter to extract the high frequency from the detail-enhanced high frequency’s source image. The two-level highpass filter will create ringing noises along with the extraction of HF information. For the LF image, we use a Gaussian lowpass filter to extract the low frequency from the source image that has a reduced dynamic range by GDC. For the extraction of the MF image, we propose a special filter that is designed as seen in Fig. 2. The magnitude can be written as

3 $$ {\mathrm{F}}_{\mathrm{M}\mathrm{F}}\left(\mathrm{D}\right)=\left\{\begin{array}{cc}\ 0,& \mathrm{D}<{\mathrm{D}}_{\mathrm{M}1}\\ {}\ 1,& {\mathrm{D}}_{\mathrm{M}1}\le \mathrm{D}<{\mathrm{D}}_{\mathrm{M}2}\\ {}\frac{1}{2}\cos \left(\frac{\uppi \left(\mathrm{D}-{\mathrm{D}}_{\mathrm{M}2}\right)}{{\mathrm{D}}_{\mathrm{M}3}-{\mathrm{D}}_{\mathrm{M}2}}\right)+\frac{1}{2},& {\mathrm{D}}_{\mathrm{M}2}\le \mathrm{D}<{\mathrm{D}}_{\mathrm{M}3}\\ {}\ 0,& {\mathrm{D}}_{\mathrm{M}3}\le \mathrm{D}\ \end{array}\right. $$

where D is the distance from the center of the filter (or zero-frequency point), and D_M1_, D_M2_, D_M3_ are the filter cutoff values.

### Local contrast enhancement

In our previous work [19], we relied on ringing generated from the HF extraction as additional noises to cover the LF part when viewed from near. However, ringing obtained by the two-level highpass filter had low contrast and gradually faded off as the distance to the original edges in the image increased. Therefore, we proposed local histogram equalization according to the location of the frequency information of the image to be paired. The map that indicates the location of the frequency information is called “local frequency map”.

In this work, we retain the use of a local frequency map for enhancing the contrast of the HF image, HF map M_HF_(**p**), using the same technique as proposed in ref. [19]. We also propose the local frequency map for the MF image as follows.

#### Local frequency map for MF image

The local frequency map for the middle frequency image determines the location of high frequency in the middle frequency image (MF map), and the location of relatively HF information in the LF image (LF map).

To know the prospective location of a particular range of frequencies in an image, it is necessary to isolate only the selected frequency band. We calculate the MF map by applying a bandpass filter to GDC (DE(I_MF_)), and the LF map by applying a bandpass filter to GDC(I_LF_). Despite the name Gaussian band pass filter, the filter shape is similar to Fig. 2, with cosine functions at both ends. There are 4 distances for determining this filter. Four parameters for determining this bandpass filter are taken from D_M1_, D_M2_, D_M3_ and HF filter size. The parameter D_M1_ indicate a starting point for the first cosine function, D_M2_ indicate its ending point where magnitude is 1 and D_M3_ indicates a starting point for decreasing cosine function and HF filter size indicates the ending point where the magnitude is 0.

Both maps are obtained by calculating the power spectral density of the specific frequency band from the bandpass-filtered image. Finally, we perform smoothing on both maps by Gaussian filter to avoid a zero-crossing position and to compute the local average. The smoothing parameters (σ) are 7 for HF map and MF map, and for LF map it is calculated from 1.2d_LF_ with LF cycle = 6, where d_LF_ is the distance for presenting LF image.

The final local frequency map for the MF image is obtained by map blending as follows:

4 $$ {\mathrm{M}}_{\mathrm{M}\mathrm{F}}\left(\mathbf{p}\right)=\left({\mathrm{k}}_{\mathrm{L}}\left(1-\mathrm{l}\left(\mathbf{p}\right)\right)+{\mathrm{k}}_{\mathrm{U}}\mathrm{l}\left(\mathbf{p}\right)\right)\left(1-\mathrm{m}\left(\mathbf{p}\right)\right)+\mathrm{m}\left(\mathbf{p}\right) $$

Here, l(**p**) and m(**p**) are the pixel values of the LF map and MF map accordingly. k_L_ and k_U_ give the lower and upper bound of the local frequency map k_L_, k_U_ ∈ [0, 1] when m(**p**) is zero (provided that l(**p**) ∈ [0, 1] and m(**p**) ∈ [0, 1]).

#### Local histogram equalization

A histogram-equalized image E_f_(**p**) at position **p** of a filtered image G_f_(**p**), f ∈ {HF, MF} is obtained by the following expression:

5 $$ {\mathrm{E}}_{\mathrm{f}}\left(\mathbf{p}\right)={\mathrm{T}}_{\mathrm{w}}\Big({\mathrm{G}}_{\mathrm{f}}\left(\mathbf{p}\right)\mathrm{c}\left(\mathbf{p}\right)+0.5\frac{1}{2}\left(1-\mathrm{c}\left(\mathbf{p}\right)\right) $$

Here, T_w_ is a transformation function of histogram equalization within a window w around the pixel **p**, and c(**p**) represents a contrast defined by the map value M_f_(**p**) as follows:

6 $$ \mathrm{c}\left(\mathbf{p}\right)={\mathrm{c}}_{\mathrm{min}}+\left({\mathrm{c}}_{\mathrm{max}}-{\mathrm{c}}_{\mathrm{min}}\right){\mathrm{M}}_{\mathrm{f}}\left(\mathbf{p}\right) $$

where c_min_ and c_max_ are user-defined values standing for the minimum and maximum contrasts. In this work, we define the same value of c_min_ and c_max_ for both the MF and HF local histogram equalization.

### Alpha compositing

The final hybrid image is obtained by combining the LF image, HF image, and MF image using alpha composition. In this work, we define the opacity values as 0.35, 0.35, and 0.3 for the HF image, MF image, and LF image, respectively.

## Results

**Fig. 3:**
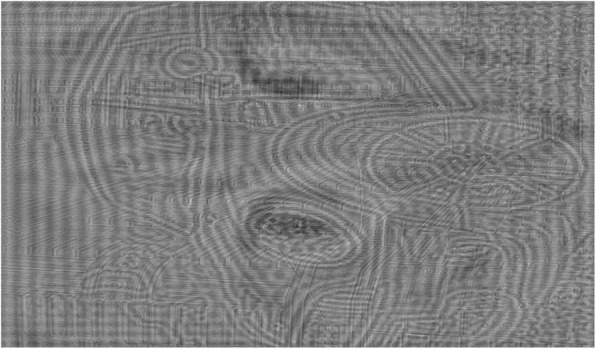
A hybrid image of a city scene, a car, and a cat to be seen from near (30 cm or 26.56° of visual angle), middle (200 cm or 4.29° of visual angle) and far distance (500 cm or 1.72° of visual angle), respectively. This figure is rotated by 90 degrees to occupy as much space as possible. Source image: “Maximum Mini”,© 2009 by Christian Senger, used under a Creative Commons Attribution license: [21], source image: “Glass Walled Building Low Angle Photography”,© 2015 by BURST, used under CC0 from https://pexels.com, and source image: “Tigger”© 2008 by Jacob Enos, used under a Creative Commons Attribution-ShareAlike license: [22]

**Fig. 4:**
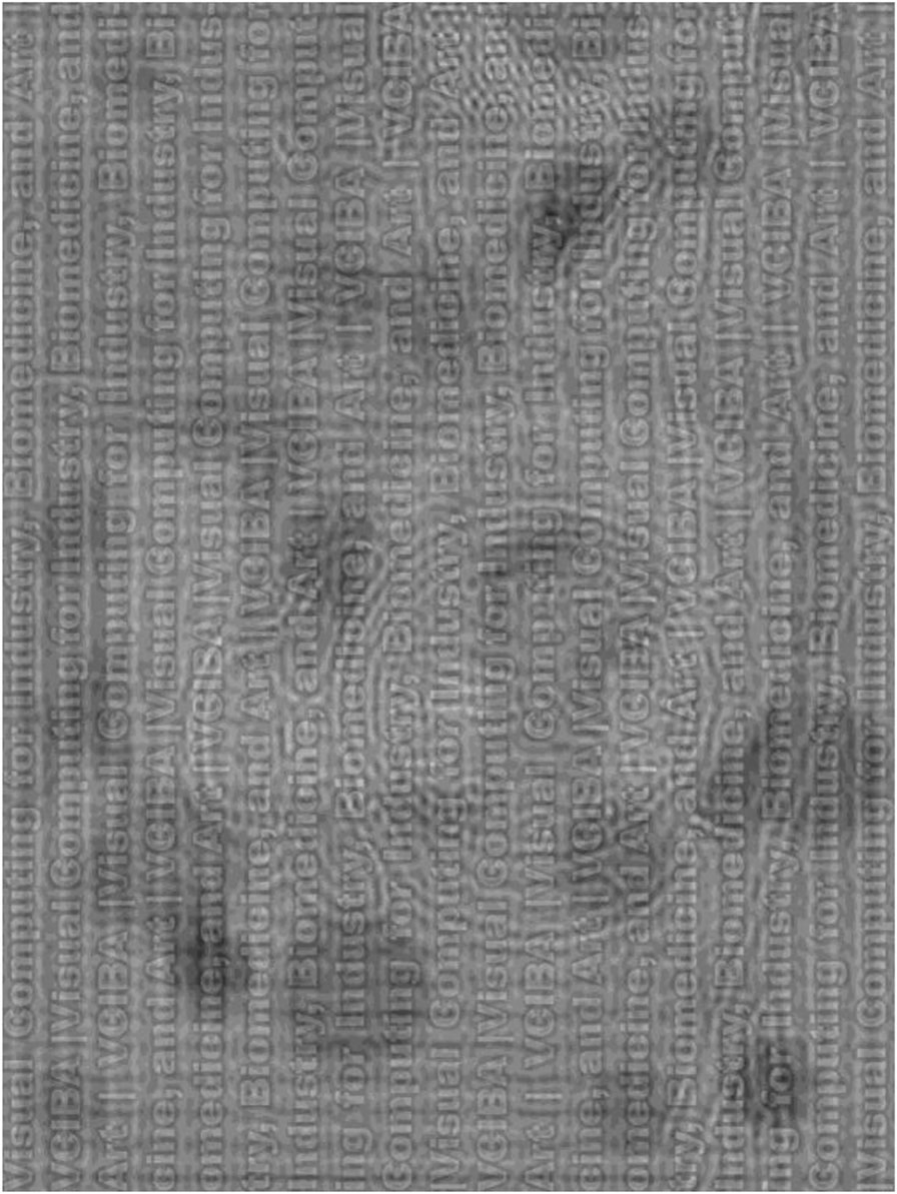
Another example of our generated hybrid image of a sequence of text, a clock on a desk, and a digit of “5” to be seen from near (30 cm or 26.56° of visual angle), middle (200 cm or 4.29° of visual angle), and far distance (500 cm or 1.72° of visual angle), respectively. This figure is rotated by 90 degrees to occupy as much space as possible. Source image for a clock on a desk: “Black Twin Bell Alarm Desk Clock on Table”,© 2017 by JESHOOTS.COM, used under CC0 from https://www.pexels.com. Other source images were synthesized by this manuscript authors

Figures 3 and 4 show the results of our proposed algorithm. The figure on an A4-size paper was calculated to be seen from three distances. The LF image was calculated to be seen from a longer distance (about 500 cm, equivalent to 1.71° of visual angle), the MF image from a middle distance (around 200 cm, equivalent to 4.29° of visual angle) and the HF image from a shorter distance (less than 30 cm, equivalent to 26.6° of visual angle). To generate the MF image, the cutoff frequency parameters for designing the special bandpass filter, D_M1_, D_M2_, and D_M3_, were 40, 56, and 120 for Fig. 3 and 40, 60, and 120 for Fig. 4, respectively. All of the input images’ sizes were 2560 × 1920 pixels.

To design the special bandpass filter for the MF image, we needed to determine suitable cutoff frequencies. In this section, we explored a range of cutoff frequencies using the same set of source images, as shown in Fig. 1.

The LF image’s cutoff frequency was fixed at σ = 16 pixels for the design of a Gaussian lowpass filter. The HF image’s cutoff frequency was fixed at 120 pixels for the design of a two-level highpass filter. The image’s size was 2560 × 1920 pixels. Therefore, the image was calculated to be viewed from a distance of less than 30 cm and more than 500 cm, displayed on a monitor size less than an A4 paper.

For the MF image’s cutoff frequencies, we divided the parameter exploration into two phases. The first phase was to test with the range of D_M1_ and D_M3_ as wide as possible, and the variability of D_M2_ determined by ratio, r. Therefore, D_M2_ could be calculated using the following equation:

7 $$ {\mathrm{D}}_{\mathrm{M}2}={\mathrm{D}}_{\mathrm{M}1}+\mathrm{r}\left({\mathrm{D}}_{\mathrm{M}3}-{\mathrm{D}}_{\mathrm{M}1}\right) $$

We tested the following ranges for the MF filter:

8 $$ {\displaystyle \begin{array}{ccc}{\mathrm{D}}_{\mathrm{M}1}& \in & \left[16,50\right],\\ {}{\mathrm{D}}_{\mathrm{M}3}& \in & \left[\mathrm{80,120}\right],\\ {}\ \mathrm{r}& \in & \left[\mathrm{0.1,0.6}\right].\end{array}} $$

From visual inspection of all generated hybrid images, we found that the lower D_M1_ was effective in making the MF image the more noticeable at a middle distance, while it was still less-noticeable when viewed from up close and far away if appropriate ringings were generated. The suitable values of D_M1_ were found to be related to the value of σ, which determines the cutoff frequency for the LF image. That is, D_M1_ should be between 2σ and 3σ.

According to the filter design in Fig. 2, D_M1_ determines the location of a sharp cutoff frequency that generates ringing for the MF image, while the location of D_M2_ indicates the size of the bandpass filter. Meanwhile, D_M3_ determines the lower base of the slope. We found that alternating D_M3_ resulted in little or no observable difference in the first experiment.

Therefore, we eliminated the parameters in the second experiment by fixing the value of D_M3_ and alternating the D_M2_ value using r. The result is shown in Table 1 with expressions and their meanings described in Table 2. From the table, we found that a D_M1_ of around 40 to 48 pixels with r between 0 and 0.3 generates a promising result. The MF image appeared noticeable in the middle distance; meanwhile, the viewer’s perception switched to the LF image when stepped away. At a closer distance, the HF image could be perceived, while the MF image appeared as a meaningless pattern.

**Table 1:** The result of phase 2 parameter tuning experiment

*D*_M1_	*r* = 0	*r* = 0.1	*r* = 0.2	*r* = 0.3	*R* = 0.4
**16**	LF < MF	LF < MF	LF < MF	LF < MF	LF < MF
	MF > HF	MF > HF	MF > HF	MF > HF	MF > HF
**24**	LF < MF	LF < MF	LF < MF	LF < MF	LF < MF
	MF > HF	MF > HF	MF > HF	MF > HF	MF > HF
**32**	LF = MF	LF = MF	LF = MF	LF = MF	LF = MF
	MF > HF	MF > HF	MF > HF	MF > HF	MF > HF
**40**	LF = MF	**LF = MF**	**LF = MF**	**LF = MF**	MF > LF
	MF > HF	**MF = HF**	**MF = HF**	**MF = HF**	MF = HF
**48**	**LF = MF**	**LF = MF**	**LF = MF**	**LF = MF**	MF > LF
	**MF = HF**	**MF = HF**	**MF = HF**	**MF = HF**	MF = HF

See Table 2 for each expression’s meaning. *HF* High frequency, *MF* Middle frequency, *LF* Low frequency

**Table 2:** Expressions and their meaning in the experiment result

Expression	Meaning
LF> MF	LF is more visible than MF
LF= MF	Either MF or LF is visible
MF> LF	MF is more visible than LF
MF> HF	MF is more visible than HF
MF= HF	Either MF or HF is visible
MF< HF	HF is more visible than MF

*HF* High frequency, *MF* Middle frequency, *LF* Low frequency

## Discussion

From the parameter tuning experiment, we found that the most critical parameter in controlling the noticeability of the MF image is D_M1_, which is the cutoff frequency that affects the size of ringing. If the generated ringing size is too coarse (fewer CPD of visual angle), the MF image will be noticeable even when the viewer steps away from the hybrid image. Meanwhile, the ringing with too much detail (more CPD of visual angle) will result in difficulty perceiving the MF image from the middle distance. In this manner, it is possible to achieve the separation of the spatial frequencies by manipulating the value of D_M1_. It could be assumed that the suitable ringing size is somehow related to CPD of the peak sensitivity in Campbell’s contrast sensitivity function [13].

**Fig. 5:**
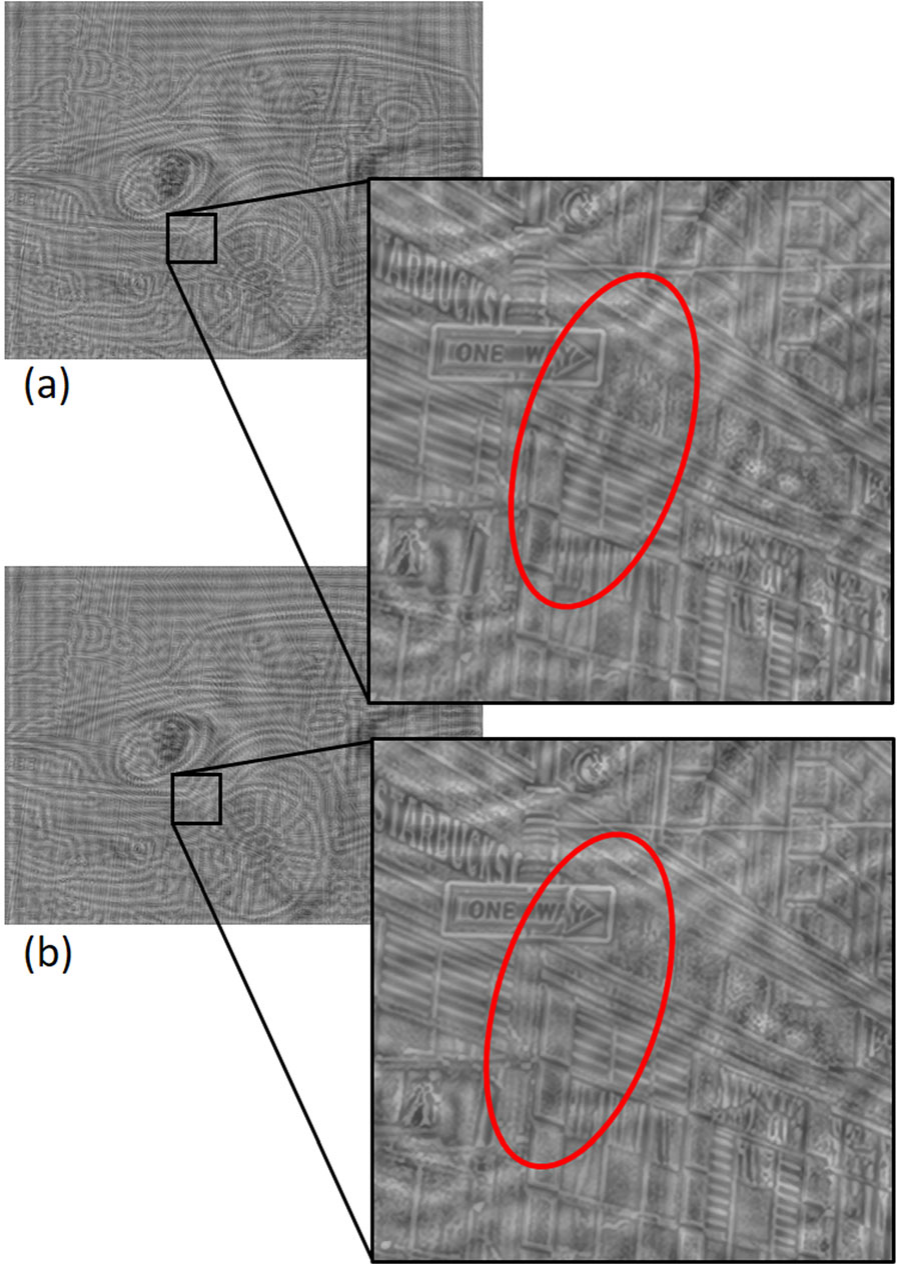
Middle frequency image’s edge continuity (**a**) and discontinuity (**b**) which may affect the perception of the middle frequency image. These phenomena are caused by parameters, D_M1, D_M2, and D_M3

In the meantime, we noticed that when the viewer was closer to the image, the MF image could be perceived as a meaningless pattern if the edges were not continuous. Fig. 5a shows the example of edge continuity, while Fig. 5b shows edge discontinuity. These phenomena may happen owing to some parameters, like D_M1_, D_M2_, and D_M3_. However, we have not figured out which parameters cause this edge continuity yet. Further investigation should be done to determine suitable parameters, including r.

## Conclusions

In this paper, we employ an adapted version of our previously proposed noise-inserted method to synthesize a hybrid image [19] from three different images. The new kind of hybrid image can be interpreted differently from three different distances; far, middle, and near viewing distances. To present one image at each distance, we use three different frequency filters, each designed to allow different frequency bands (the low, middle, and high) to pass. We propose a special bandpass filter (MF filter) for extracting frequencies to be seen from the middle distance. To determine the suitable cutoff frequencies for designing the MF filter, we conducted a parameter tuning experiment. As a result, we found that a suitable parameter for D_M1_ is linked to the σ for the LF filter. Meanwhile, the determination of suitable values for other parameters requires further investigation. In the future, we plan to conduct an experiment to measure the separation of the spatial frequencies when viewing the hybrid image from three different distances.

## Data Availability

The datasets generated and/or analyzed during the current study are available in the Google Drive repository (https://drive.google.com/drive/folders/1yS__xWBEzcXOd3SYiW2lhmskTY41bdty?usp=sharing).

## Abbreviations

CPD: Cycle per degree
DE: Detail enhancement
GDC: Gradient Domain image range Compression
HF: High frequency
LF: Low frequency
MF: Middle frequency
