# Correction to: Hybrid image of three contents

**DOI:** 10.1186/s42492-020-00046-w

**Published:** 2020-03-24

**Authors:** Peeraya Sripian, Yasushi Yamaguchi

**Affiliations:** 1 SIT Research Laboratory, Shibaura Institute of Technology, Room# 04R31, 3-7-5 Toyosu, Koto-ku, Tokyo, 135-8548 Japan; 2 The University of Tokyo, Bldg. #15, Room #505A, 3-8-1, Komaba, Meguro-ku, Tokyo, 153-8902 Japan

**Correction to: Vis Comput Ind Biomed Art (2020) 3:4**

**https://doi.org/10.1186/s42492-019-0036-3**

In the original publication of this article [1], the Figs. 3 and 4 are not clear enough. They are adjusted the size and showed as below:

**Fig. 3:**
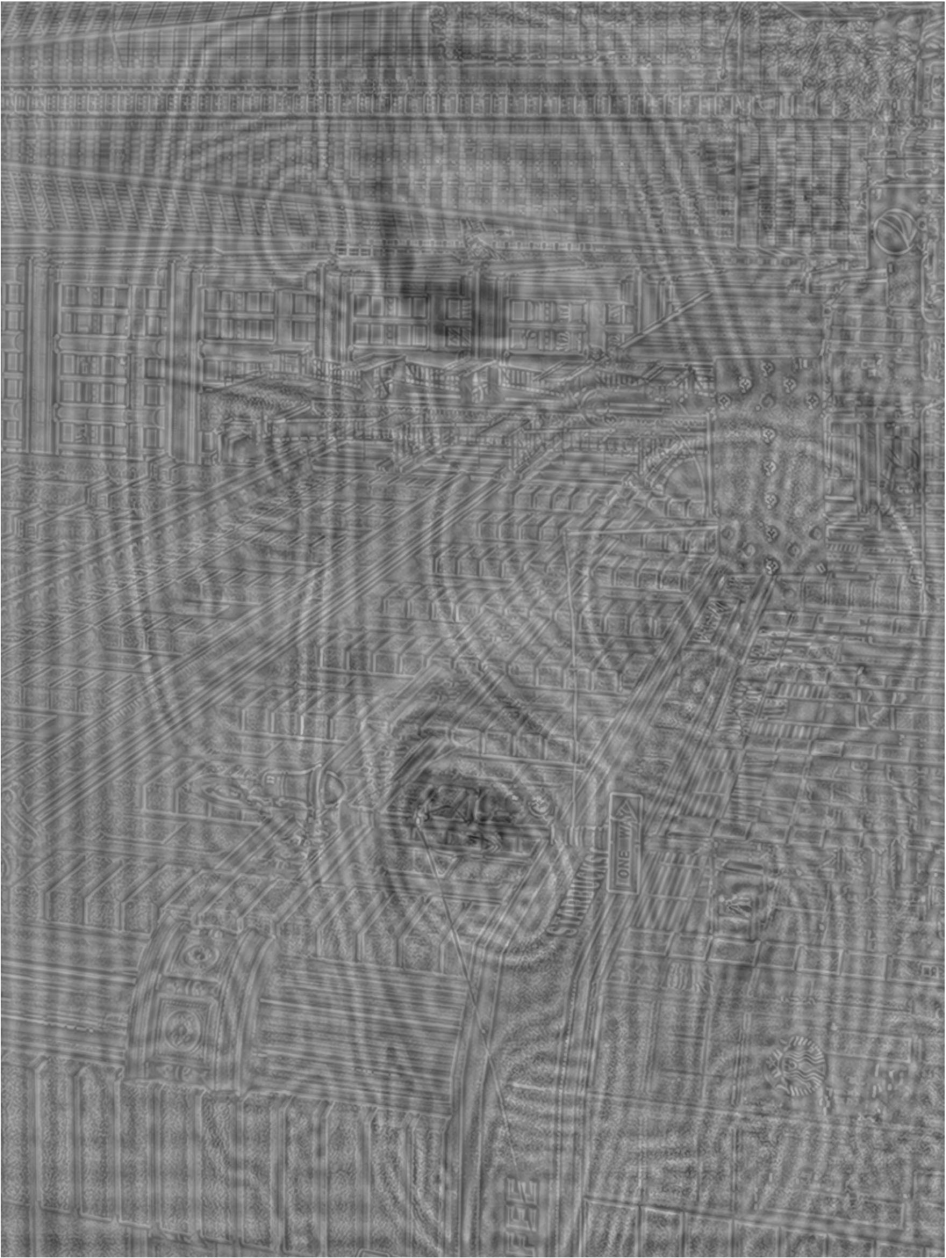
A hybrid image of a city scene, a car, and a cat to be seen from near (30 cm or 26.56° of visual angle), middle (200 cm or 4.29° of visual angle) and far distance (500 cm or 1.72° of visual angle), respectively. This figure is rotated by 90 degrees to occupy as much space as possible. Source image: “Maximum Mini”,© 2009 by Christian Senger, used under a Creative Commons Attribution license: [21], source image: “Glass Walled Building Low Angle Photography”,© 2015 by BURST, used under CC0 from https://pexels.com, and source image: “Tigger”© 2008 by Jacob Enos, used under a Creative Commons Attribution-ShareAlike license: [22]

**Fig. 4:**
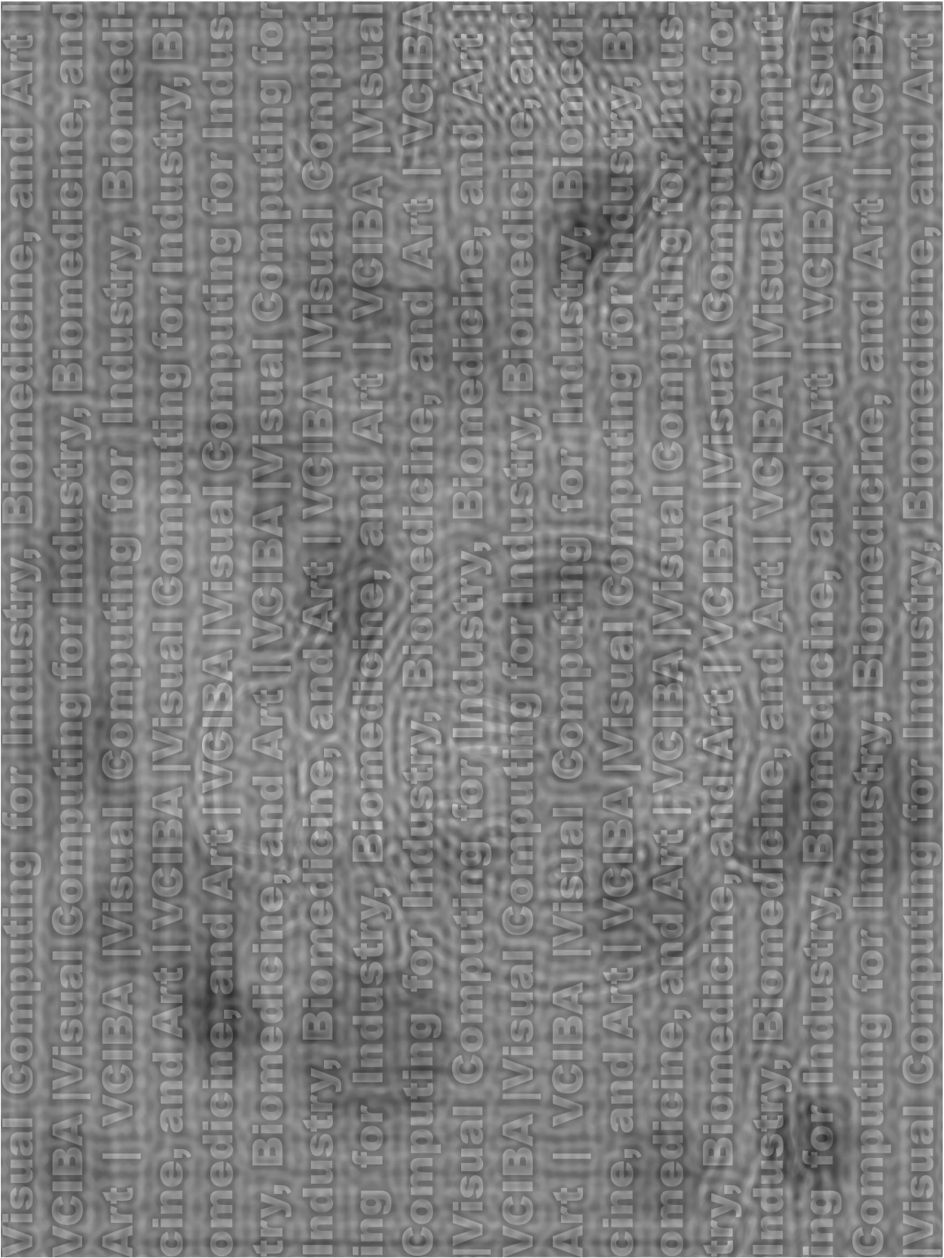
Another example of our generated hybrid image of a sequence of text, a clock on a desk, and a digit of “5” to be seen from near (30 cm or 26.56° of visual angle), middle (200 cm or 4.29° of visual angle), and far distance (500 cm or 1.72° of visual angle), respectively. This figure is rotated by 90 degrees to occupy as much space as possible. Source image for a clock on a desk: “Black Twin Bell Alarm Desk Clock on Table”,© 2017 by JESHOOTS.COM, used under CC0 from https://www.pexels.com. Other source images were synthesized by this manuscript authors
